# Flexural Properties and Low-Velocity Impact Behavior of Polyamide 11/Basalt Fiber Fabric Laminates

**DOI:** 10.3390/polym13071055

**Published:** 2021-03-27

**Authors:** Libera Vitiello, Pietro Russo, Ilaria Papa, Valentina Lopresto, Davide Mocerino, Giovanni Filippone

**Affiliations:** 1 Department of Chemical, Materials and Production Engineering, University of Naples Federico II, Piazzale Tecchio 80, 80125 Naples, Italy; ilaria.papa@unina.it (I.P.); lopresto@unina.it (V.L.); davide.mocerino@unina.it (D.M.); gfilippo@unina.it (G.F.); 2 Institute for Polymers, Composites and Biomaterials–National Council of Research, 80078 Pozzuoli (Naples), Italy

**Keywords:** polyamide 11, basalt fiber, biocomposite, flexural properties, low-velocity impact

## Abstract

Environmentally friendly composite plates intended for load-bearing applications were prepared and systematically characterized in terms of mechanical performances and morphological features. Sample plates combining two extrusion grades of bio-polyamide 11, one of which is plasticized, and two basalt fiber fabrics (plain weave and twill architectures) were obtained by film stacking and hot pressing, and their mechanical properties were investigated by quasi-static flexural and low-velocity impact tests. The comparative analysis of the results, also interpreted by the bending damage analysis, through optical microscope observations, and impact damage analysis through visual inspection and indentation measurements demonstrate that, besides interfacial adhesion issues, the mechanical performance of the laminates need to be optimized through a careful selection of the constituents in the light of the final application. In particular, if the goal is a gain in impact strength, the use of the plasticized matrix is beneficial, but it brings about a loss in stiffness and strength that can be partially compensated by properly selecting a more performing fiber fabric architecture. The latter must also be easily permeated by the matrix to enhance the efficiency of stress transfer from the matrix. Overall, our results can be exploited for the development of bio-composites for particularly demanding applications.

## 1. Introduction

In the last decades, basalt, initially widely used exclusively for aeronautical and defense applications, has been of extraordinary interest in the development of polymeric composites, especially in the form of reinforcing fibers as a valid alternative to conventional glass ones [1]. Subramanian and Austin [2] were the first to report the role of interfacial adhesion of pre-functionalized basalt fibers with silane groups in thermosetting polyester matrix composites. 

Recently, in the context of the growing attraction towards thermoplastic composites able to combine structural performance, recyclability, and lightness, many research efforts dealing with basalt-reinforced materials have been published [3]. Currently, experimental evidence is available on the preparation and characterization of many thermoplastic polymers, such as polypropylene [4,5], polyethylene [6,7], polyamides [8,9], poly(ethylene terephthalate) [10,11], and poly(lactic acid) [12,13], including basalt fibers. In this wide range of research works, the use of polyamides stands out for their polar structure potentially capable of giving rise, in combination with basalt fibers, to composites with a discrete interfacial affinity [14]. Prajapati et al. [15] investigated the mechanical and morphological properties of melt-mixed nylon 6 composites comprising different amounts of both chopped basalt fibers and a coupling agent (polyethylene-grafted-maleic anhydride, PE-g-MA). They demonstrated relevant improvements of mechanical properties for samples modified with this latter additive. 

Lee et al. [16] reported on the influence of fiber length and orientation and wax-based compatibilizing agent on the mechanical properties of polyamide 6 composites. Experimental tests demonstrated the benefits of the compatibilizing agent on the thermomechanical properties of PA6 composites, while the numerical analysis highlighted an increase in the elastic modulus of the materials and a loss of orientation of the basalt fibers as their length increases.

Yu et al. [17] developed high-strength and lightweight polyamide 6,6 composites filled with plasma-treated basalt fibers to improve the interfacial adhesion. In more detail, authors considered basalt fibers exposed for different lengths of time to a plasma, using 3-aminopropyltriethoxysilane (APTES) as a precursor for their surface modification. This study demonstrated the formation of a thick polymeric layer compatible with the PA6.6 polymeric matrix on the surface of the basalt fibers and, consequently, a significant improvement in the performance of the composites obtained with these fibers compared to those containing a similar content of neat basalt ones. 

Deak et al. [18] focused their attention on the surface modification of basalt fibers with the use of silanes typically employed for glass fibers. The inclusion by melt-compounding of basalt fibers, preliminarily immersed in a solution of different coupling agents, in a PA6 matrix provided samples of composites with different reinforcement contents. The mechanical analysis by means of static and dynamic tests highlighted a significant increase in mechanical performance, with more marked effects in the case of basalt fibers coated with 3-glycidoxipropyltrimethoxysilane. 

In a scenario of research and industrial interests, ever more widely addressed to structural or semi-structural thermoplastic composites, worthy of note are further efforts and experimentation dealing with the influence of the matrix structure (degree of crystallinity, presence of plasticizers) on the ultimate mechanical properties of these materials [19,20]. Together with a multitude of other factors already mentioned above, such as interfacial adhesion and fiber orientation, this issue plays a key role and must be taken into account in optimizing the process conditions in order to obtain products meeting specific market needs. In this regard, the results obtained so far about the effect of structural issues on the ultimate performance of composite products are often contradictory. For example, regarding the influence of crystallinity on the mechanical properties of thermoplastic laminates, starting from the assumption that the actual degree of crystallinity of the matrix is dependent on the cooling rate [21,22], some studies have indicated an increase of the interfacial shear values with an increasing cooling rate for polypropylene/glass fiber laminates [23], while other research has showed that this influence is opposite for carbon fiber/polypropylene composites [24], or even almost insignificant for carbon fiber/polyethylene terephthalate systems [25].

Plasticizers are usually added to thermoplastic matrices to improve their flexibility and toughness. Woigk et al. [26] investigated thermoplastic composite laminates combining cellulose propionate films with a unidirectional flax fiber fabric via a film stacking and hot pressing procedure. The matrix was preliminarily modified by adding 10 wt.% of a commercial plasticizer and 2 wt.% of a suitable slip agent to improve its processability and wetting behavior. The results showed a significant improvement in mechanical properties, with a 90% increase in notched strength compared to similar samples with an unmodified matrix.

However, Oksman et al. [27], studying flax-fiber-reinforced composites based on polylactic acid, preliminarily modified with triacetin as a plasticizing agent, showed an improved processability of the matrix, but, contrary to expectations, no benefit in terms of improvement of the impact properties of the products.

In other words, it is not possible to draw generalized considerations about the effect of the structure of the matrices on the final performance of thermoplastic composites, and, from time to time, appropriate investigations are mandatory, especially in the case of the development of innovative thermoplastic systems.

Over time, despite the significant mechanical performance and potential use of products containing basalt, up to now developed at least on a laboratory scale, a further drift of the research has been strongly conditioned by the need to reduce the environmental impact of the reference products at the end of their useful life. For this reason, intense experimentation has been focused on the use of bio-based polymeric matrices as sustainable alternatives to oil-based ones. With reference to polyamides, the use of resins such as polyamide 610, polyamide 1012, and polyamide 11, all obtained partially or entirely from castor oil, has been explored. Among these, polyamide 11 is not biodegradable and has been already validated for durable engineering applications in various sectors such as automotive, biomedical, and sports. In this case, PA11 composites have already been the subject of studies [28,29,30,31,32], but, to the best of our knowledge, no data on the performance of PA11/basalt composites are available so far. That said, taking into account the inevitable technological challenges induced by the use of biomaterials on an industrial scale, and with the aim of further investigating the reinforcement and damage mechanisms triggered in bio-composites, this research work deals with composite systems obtained by combining two commercial PA11 bio-resins and two basalt fiber fabrics with different architecture. The laminates prepared by film stacking and hot pressing were systematically characterized with quasi-static bending tests and dynamic low-velocity impact tests. The analysis of both mechanical results and damage with visual inspections and indentation measurements, as well as morphological observations, can provide a useful advancement of knowledge to better appreciate the potential uses of the bio-composites studied.

## 2. Materials and Methods

### 2.1. Materials

Investigated materials are based on two bio-based polyamide 11 matrices supplied by Archema S.A. (Colombes, France) under the trade names RILSA^®^ BESNO TL and RILSAN^®^ BESNO P40. In particular, the former grade, designed for extrusion, have a content of renewable carbon higher than 98%, according to ASTM D 6866, and it is characterized by, among other things, density ρ = 1.03 g/cc, melting temperature Tm = 189 °C, and melt flow index MFI = 11 cc/10 min at 235 °C/2.56 kg. The BESNO P40, on the other hand, is a plasticized extrusion grade including 12 wt.% of benzene-butylsulfonamide. Some physical properties of this matrix are the following: ρ = 1.05 g/cc, Tm = 180 °C, and MFI = 3.72 g/10 min at 235 °C/2.56 kg. The reinforcement of these polymeric matrices was made by including basalt fiber fabrics with two different architectures: plain weave (BAS 220.1270.P) and 2 × 2 twill (BAS.220.1270.T), both with the same areal weight of 220 g/m^2^ and both supplied by Basaltex (Wevelgem, Belgium).

### 2.2. Composite Fabrication

Preliminarily, bio-polyamide 11 resins were dried in a vacuum oven at 70 °C overnight, and transformed in 75–80 μm thick films by a flat die extruder Teach-Line E 20-T equipped with a calender CR 72T Collin (Maitenbeth, Germany), using a temperature profile of 170°—210°—220°—220°—200°, from the hopper to the die, and a screw speed of 60 rpm. 

**Figure 1:**
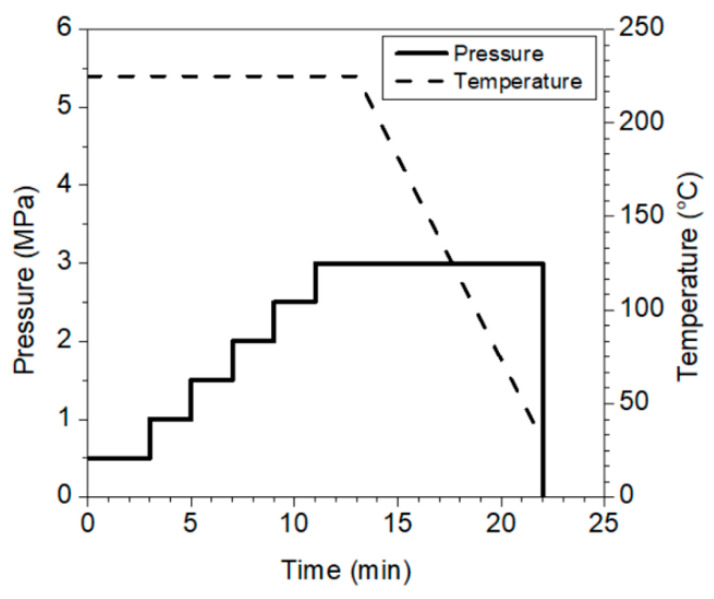
Processing conditions for the preparation of all basalt-reinforced laminates.

Composite laminates were prepared by alternately superimposing bio-polyamide 11 film and basalt fabric layers in accordance with a specific previously established sequence, and then consolidated by hot pressing using a lab-press Collin P400E (Maitenbeth, Germany) according to pre-optimized processing conditions sketched in Figure 1, regardless of the degree of PA11 and the architecture of the reinforcement fabric considered. The number of superimposed layers (i.e., the number of constituent plies) for each laminate was such as to have samples with a thickness approximately equal to 3 mm. By operating in this way, square plates of four samples, each with 320 mm sides and reinforcement volume contents of about 45% (ASTM D3171), were obtained, which are coded hereafter as Plain_TL, Plain_P40, Twill_TL, and Twill_P40.

### 2.3. Experimental Techniques

Quasi-static flexural tests were conducted, adopting a three-point configuration, according to ASTM D790-07 Standard. Specimens of 80 × 13.5 × 3 mm^3^ cut from each laminate were subjected to a concentrated load applied at a speed of 1.7 mm/min along both the width and the centerline. These determinations, which were carried out on at least five specimens for each sample with the aid of an MTS Alliance RT/50 dynamometer (Minneapolis, MN, USA) equipped with a 50 kN load cell, made it possible to derive typical stress-strain curves. Processing of these provided some mechanical parameters, such as modulus and flexural strength, reported later in terms of mean values and standard deviations.

Low-velocity impact tests were performed using a Ceast Fractovis (Pianezza, Torino, Italy) falling dart machine equipped with an instrumented impactor with a mass of 3.64 kg and a hemispherical nose, which was 19.8 mm in diameter. On the basis of previous impact tests, the impact energy was set at 200 J to obtain a complete penetration of each 100 × 150 mm^2^ specimen. Further, the so-called indentation measurements were performed at lower impact energies (10 J, 20 J, 30 J) on square 100 × 100 mm^2^ specimens to monitor the evolution of the damage. In this case, the reproducibility of the results was ascertained with at least three tests in the same conditions.

The cross-sections of specimens were inspected with a TM 3000 field emission scanning electron microscope (Hitachi High-Technologies Corporation, Tokyo, Japan) operating in high vacuum conditions at the voltage of 20 kV. The specimens were sputter-coated with a thin layer of a gold–palladium alloy prior to SEM observations. All the SEM images were obtained using back-scattered contrast.

## 3. Results and Discussion

### 3.1. Flexural Properties

**Figure 2:**
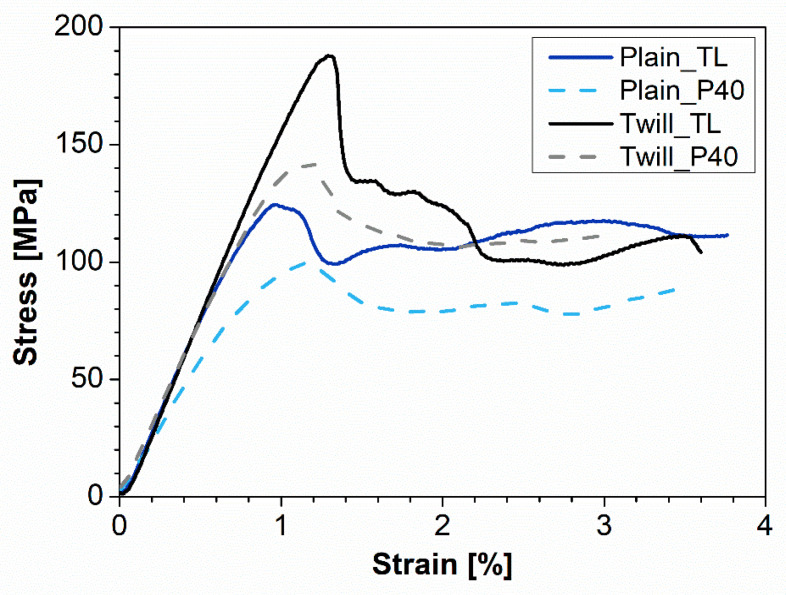
Representative flexural stress-strain curves for all investigated materials.

Figure 2 compares the flexural behavior of all manufactured samples. Data obtained by processing these curves are collected in Table 1 in terms of mean values and standard deviations of flexural modulus and strength. 

**Figure 3:**
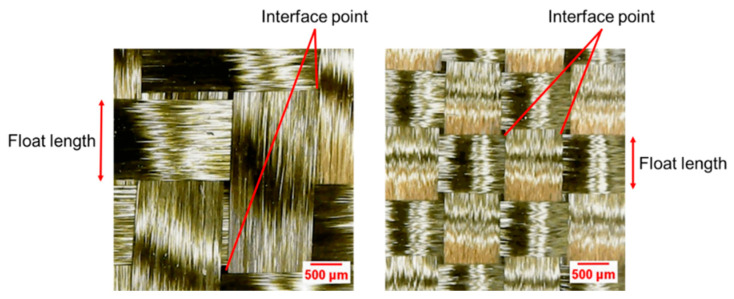
Twill (**left**) and plain (**right**) weave structures.

Clearly, higher flexural parameters are obtained in the presence of the twill basalt fiber fabric. This result is perfectly in line with the awareness that, despite the nature of woven fibers, twill-fabric-reinforced specimens show superior performances in comparison with plain weave fabric ones [33]. This peculiarity, widely consolidated in the literature, is explained by considering that twill weave architecture is characterized by longer float length and higher fiber bundle number per unit cell than plain weave. Furthermore, the plain weave has a tight pattern, high crimp angle, and high interlace point, which result in a non-negligible void content. On the contrary, the twill weave has a loose pattern, less crimp angle, and fewer interlace points (Figure 3). 

**Figure 4:**
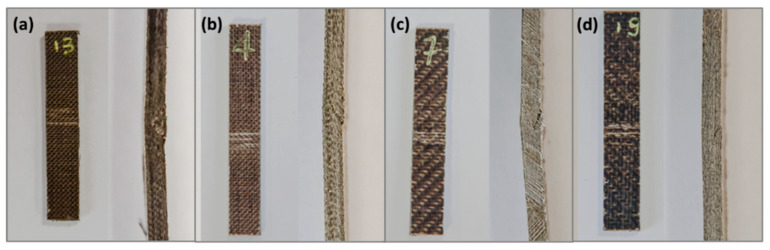
The specimens tested with a bending test: (**a**) Plain_TL, (**b**) Plain_P40, (**c**) Twill_TL, and (**d**) Twill_P40.

Furthermore, with the same architecture of the reinforcement, the composites based on the plasticized matrix (P40) show a greater ductility than those obtained starting from the TL matrix. This behavior is, all in all, expected, thanks to the greater flexibility of the polymer chains induced by the presence of a plasticizing additive, and is reflected in a greater deformation at break. It can also be partially attributed to a greater physical interfacial interaction with the reinforcing fibers, generated by the higher viscosity of the plasticized bio-polyamide compared to the non-plasticized one. Pictures collected in Figure 4 highlight a compression instability for all investigated samples, with clear fiber debonding phenomena in correspondence with the applied bending stress probably linked to the poor interfacial chemical affinity between the host matrix and the reinforcing fibers.

**Figure 5:**
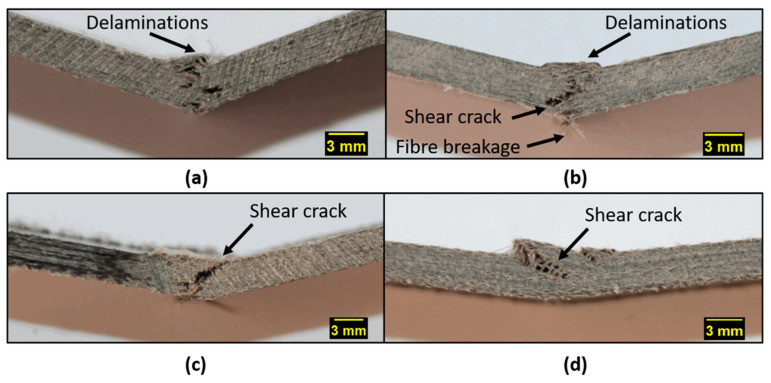
Optical through-thickness micrographs corresponding to the bending point: (**a**) Plain_TL, (**b**) Plain_P40, (**c**) Twill_TL, and (**d**) Twill_P40.

A further study of the damage mechanisms that occur under bending is provided by the analysis of the microscopic observations, which were collected through the thickness of the tested specimens at the point of application of the load (Figure 5) by a Canon Eos 1200D equipped with a lens of 55 mm. As established, a composite laminate subjected to bending can fail due to fiber breakage, matrix cracking (shear cracks), and/or by the onset and growth of interfacial delamination [34,35]. In our case, apart from the instability phenomena already mentioned, the prevalence of shear crack phenomena is evident for the twill-basalt-fabric-based system, confined to the compression region for the PA11-P40 specimens, and extended along the entire thickness for the PA11-TL ones. On the contrary, for the specimens reinforced with plain architecture fabric, the delamination phenomena seem to dominate the flexural damage, with a more pronounced effect for the systems with non-plasticized matrixes (Plain_TL). In particular, both delamination in the compression side and matrix cracking in the tensile portion of the specimen seem to occur for Plain_P40 specimens, which even show fiber failure signs on the side of the specimen opposite to the bending point. 

Given that the onset and propagation of delamination is strictly related to the interfacial fiber–matrix interaction [36], these observations confirm a greater fiber–matrix interaction in twill laminates compared to plain weave ones. Moreover, for systems containing twill basalt fabric layers, the plasticized matrix ensures that the failure of the matrix remains limited to the compressed region of the specimen, due to its expected ductility manifested mainly in the region of the specimen subject to tensile stresses.

### 3.2. Low-Velocity Impact Behavior

Table 2 summarizes the mean value and the standard deviation of some impact parameters obtained from the processing of the typical force-displacement curves detected with the penetration tests. Specifically, the systems studied are compared in terms of maximum contact force (F_max_) and displacement (d) corresponding to this maximum force—that is, in other words, corresponding to the incipient failure of the specimen and penetration energy (U_p_).

Clearly, for systems with plain reinforcement, the maximum shape assumes a higher average value in the presence of the non-plasticized matrix, while the opposite occurs for laminates with reinforcement that have a twill architecture. This effect, taking into account the scarce chemical interaction between matrix and reinforcement, can be explained by assuming, in the first case, the influence of the greater rigidity of the PA11_TL matrix, and, in the second case, the greater capacity of the plasticized matrix to wrap on a reinforcement capable of ensuring greater interfacial physical interaction.

Regarding the displacement at the maximum contact force, it is evident that, regardless of the reinforcement’s architecture, this parameter assumes higher mean values for composites with a plasticized matrix than those based on the non-plasticized one. This behavior, in line with the penetration energy trend, can be attributed to a greater ability for plastic deformation induced by a plasticizing agent in the matrix.

**Figure 6:**
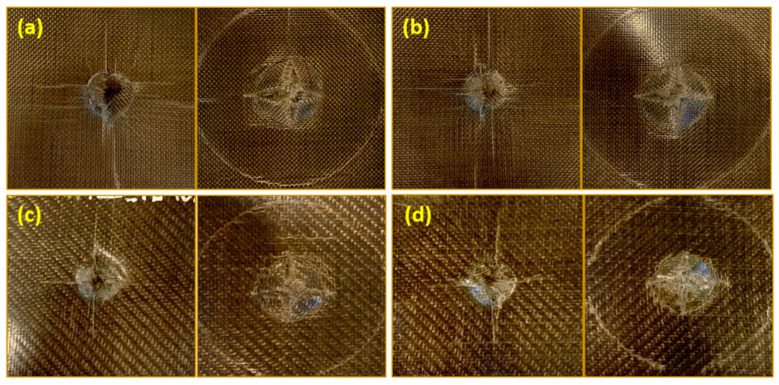
Pictures of the front (left) and rear (right) surfaces of the impacted Plane_TL (**a**), Plane_P40 (**b**), Twill_TL (**c**), and Twill_P40 (**d**) specimens.

These results are also highlighted by the front and rear pictures of the plain and twill weave (Figure 6) basalt-fabric-reinforced systems. In general, matrix type and fabric architecture are key factors, among others, affecting damage mechanisms and extent. In our cases, a circular shape of the damage is always observed with a footprint that, with the same extension and resolution of the image, appears darker for the plasticized PA11-based specimens. This optical effect—reasonably attributable to a greater propagation of damage along with the thickness of the specimen, given the greater plastic deformation capacity of this matrix compared to the non-plasticized one—is, moreover, supported by the detection of a greater displacement in correspondence with the maximum contact force (see Table 2). Furthermore, for all the penetrated specimens, a cross-shaped crack path along the warp and weft directions of the basalt fabric is evident on the rear (non-impacted) surface. 

**Figure 7:**
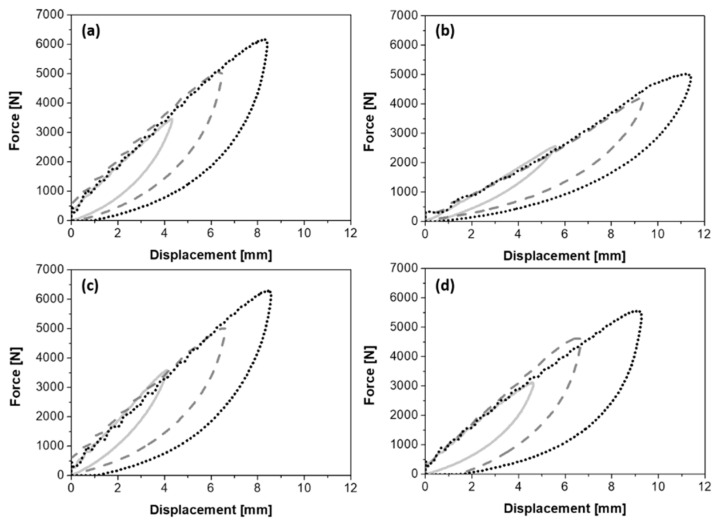
Load-displacement curves at indentation for the laminates Plain_TL (**a**), Plain_P40 (**b**), Twill_TL (**c**), Twill_P40 (**d**) at three impact energy levels: 10 J (solid line), 20 J (dashed line), and 30 J (dotted line).

**Figure 8:**
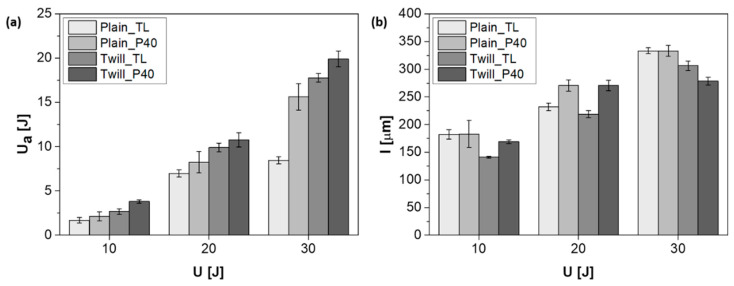
Absorbed energy (**a**) and indentation depth (**b**) as a function of the impact energy for all investigated materials.

Regarding the indentation measurements, Figure 7 shows the representative load-displacement curves of plain and twill weave basalt-fiber-fabric-reinforced samples with and without the plasticizer and collected after impacting at three energy levels (10 J, 20 J, and 30 J). The indentation parameters are partially collected in Table 3, which reports the average values and standard deviations of the maximum force (F_max_) and the corresponding displacement (d) for each impact energy (U). At the same time, special attention is paid to the absorbed energy (U_a_) and the indentation depth (I), whose values were better highlighted with the histograms of Figure 8a,b.

Clearly, despite the expected increase of the maximum impact load with the increase of the impact energy, the plasticizing agent’s presence affects the F_max_ parameter. In the same way, a higher value of displacement at the fibers’ break is achieved by plain weave laminates compared to the twill one, especially in the case of the plasticized matrix.

The plasticization of the matrix also generates an increase of the absorbed energy with effects, also in this case, more pronounced in the presence of a twill-type reinforcement fabric architecture. In general, the greater impact benefits found for composites reinforced with a twill fabric could be attributed to a greater physical interaction of this architecture with the surrounding matrix [37]. The results indicated that under the same impact energy, the energy absorbed and therefore the damage suffered by the composites with the plasticizing agent is greater than in the case of the non-plasticized ones (see Figure 8a).

**Figure 9:**
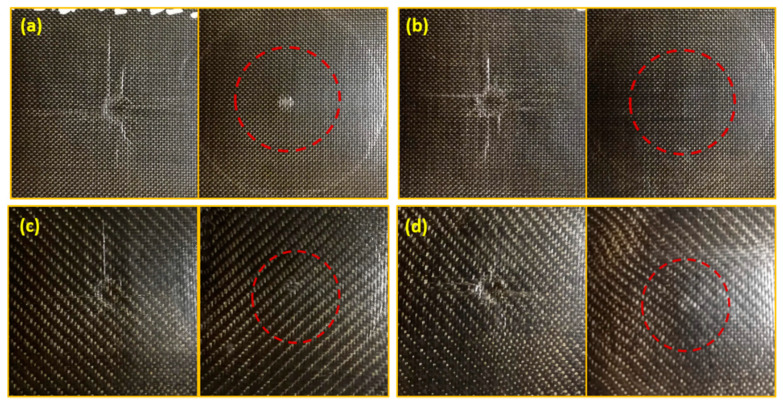
Visual inspections of front (left) and rear (right) surfaces of Plane_TL (**a**), Plane_P40 (**b**), Twill_TL (**c**), and Twill_P40 (**d**) samples; U = 30 J.

In this regard, focusing on the specimens impacted at the 30 J energy level, Figure 9 reports the observations of the visual inspection performed on their front and rear (non-impacted) surfaces.

For composites based on the plasticized PA11 and reinforced with plain weave basalt fabric, a reduction in plastic deformation or indentation (red circles in Figure 9a,b) is noted, being the footprint impressed by the impactor on the impacted side. Probably, the higher absorbed energy evaluated for this system (see Figure 8a) is mostly spent for delamination in the plane, rather than for plastic deformation. In other words, the use of plasticized matrices appears to bring effects similar to those obtained by increasing the thickness of the tested specimen under the same conditions. The same seems to happen in the Twill_P40 system, even if its results are less evident (red circles in Figure 9c,d). The evaluation of the indentation depth (see Figure 8b) partially confirms these findings. This parameter, representing the footprint impressed by the impactor on the impacted surface of the samples, grows with the impact energy; however, in our cases, it is also influenced by both the plasticization of the matrix and the extent of the interfacial interactions. That said, given that the complex relationship between all these variables makes the trends of the indentation depth unpredictable, with specific reference to the experimental campaign carried out so far, it can be stated that, while at low energy levels (10 J and 20 J), regardless of the architecture of the basalt fabric, the presence of the plasticizing agent favors a greater plastic deformation of the specimens. This effect is apparently cancelled and even reversed for impacts at 30 J. This behavior, although it needs to be validated by a greater number of tests and non-destructive investigations of the internal damage, is obviously a sign of the triggering of various damage mechanisms [38,39]. For the Twill_P40 system, given the greater capacity of the fabric to provide fiber–matrix physical interactions and the greater deformability of the plasticized PA11, it is reasonable to assume that a large portion of the energy, absorbed by this system for impacts at 30 J, is mainly spent on fiber fracture phenomena, rather than for external permanent deformations.

### 3.3. Morphological Analysis 

**Figure 10:**
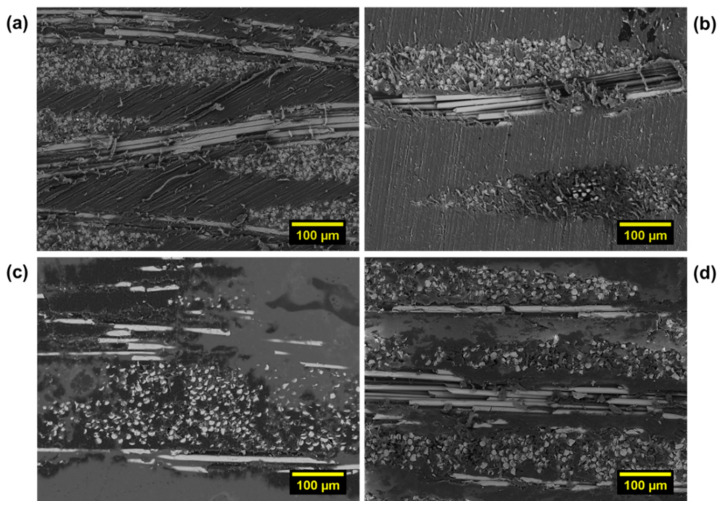
Representative SEM micrographs (magnification: 300×) of cryo-fractured surfaces: (**a**) Plain_TL, (**b**) Plain_P40, (**c**) Twill_PL, and (**d**) Twill_P40.

Finally, representative micrographs of cryo-fractured surfaces of all investigated composite systems are compared in Figure 10. Examination of the morphological aspects confirms the following:

• Regardless of the type of architecture of the reinforcement fabric, the plasticized matrix P40 wraps the reinforcement fibers better than the not-plasticized PA11 TL grade.
• On the other hand, with the same type of matrix, the twill fabric guarantees a better interfacial interaction than the plain architecture. Moreover, an indirect indication of a better impregnation achieved in the twill samples is provided by the lower polymeric matrix presence between the layers. In this case, the polyamide 12 flows more easily in the fiber bundles due to the fabric’s architecture.

These considerations are in line with mechanical behavior and damage analysis previously reported and discussed.

## 4. Conclusions

Composite plates based on two commercial PA11 bio-polyamides (pure and plasticized) and two basalt fiber fabrics with the same areal weight but different architectures (plain weave and twill) have been manufactured, and characterized in terms of mechanical performances, morphological issues and damage analyses. In particular, specimens cut from the laminates were characterized by quasi-static flexural and low-velocity impact measurements, and the collected results were explained in light of SEM observations, visual inspections, and indentation measurements. The experimentation highlighted the significant influence of the ductility of the matrix and the architecture of the reinforcement on the composite’s final properties. Specifically, the data showed that the presence of plasticizer in the matrix contributes to a greater deformation and energy absorption capacity of the composite, with a slight reduction in mechanical stiffness. This reduction in mechanical stiffness, however, can be compensated by using a more performing reinforcement architecture, such as the twill compared to the plain weave one. In fact, typical features of the twill basalt layers provide higher physical interactions with the surrounding matrix, as also witnessed by the through-thickness optical microscopy observations in correspondence with the bending point, and by SEM morphological analysis of the sections of each sample studied. Finally, considering the experimental findings collected in this research work, useful information can be drawn for the development and optimal design of environmentally sustainable composites to validate their use, even in more advanced engineering applications.

## Figures and Tables

**Table 1:** Mean values of flexural modulus (E_f_) and maximum flexural strength (σ_f_) for each sample type. Standard deviations are reported in brackets.

Sample Type	E_f_ [GPa]	σ_f_ [MPa]
Plain_TL	12.8 (1.8)	124 (17)
Plain_P40	11.0 (1.2)	91.5 (12)
Twill_TL	15.7 (1.1)	174 (25)
Twill_P40	14.8 (1.0)	140 (19)

**Table 2:** Impact parameters at penetration. Standard deviations are reported in brackets.

Sample Type	F_max_ [N]	d [mm]	U_p_ [J]
Plain_TL	9464 (200)	14.1 (2.5)	131.6 (10.5)
Plain_P40	9189 (100)	16.4 (0.9)	152.2 (4.5)
Twill_TL	8680 (176)	12.2 (1.7)	106.7 (12.3)
Twill_P40	9797 (185)	14.8 (2.1)	132.9 (14.0)

**Table 3:** Impact parameters at indentation for all the samples. Standard deviations are reported in brackets.

Sample Type	U [J]	F_max_ [N]	d [mm]
Plain_TL	10	3592 (85)	4.3 (0.9)
	20	5074 (78)	6.6 (0.5)
	30	6165 (77)	8.5 (1.0)
Plain_P40	10	2934 (120)	5.6 (1.2)
	20	4197 (100)	9.4 (1.5)
	30	5016 (95)	11.4 (0.9)
Twill_TL	10	3807 (84)	4.1 (0.6)
	20	5035 (80)	6.6 (0.8)
	30	6296 (66)	8.5 (0.5)
Twill_P40	10	3259 (95)	4.6 (0.6)
	20	4911 (90)	6.7 (0.5)
	30	5558 (60)	9.1 (0.2)
